# Supplementary figures and images for: HBZ upregulates myoferlin expression to facilitate HTLV-1 infection
Source: PLoS Pathog. 2023 Feb 24;19(2):e1011202. doi: 10.1371/journal.ppat.1011202 (PMC9994761; doi:10.1371/journal.ppat.1011202)

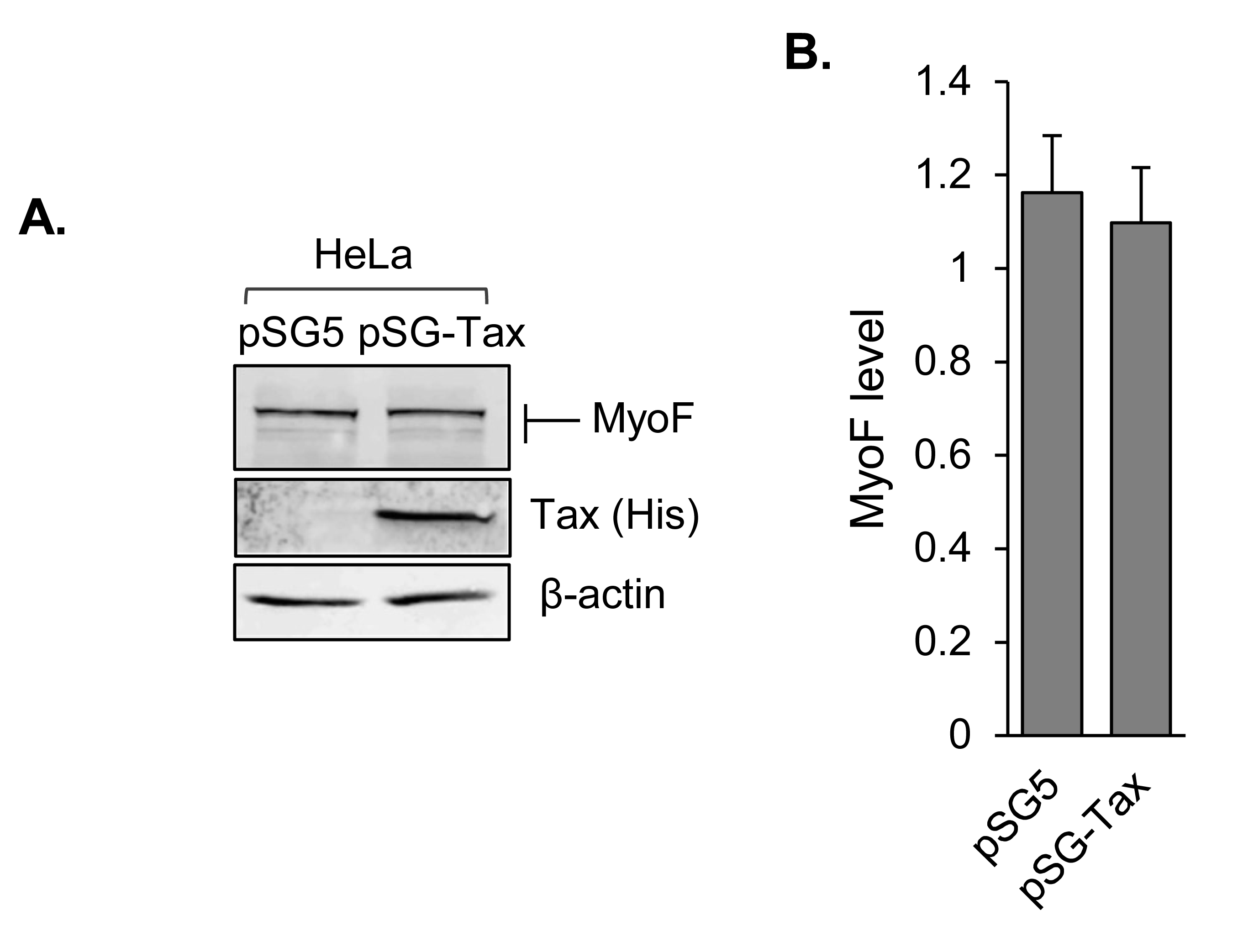

Supplement: S1 Fig — (A) MyoF expression in HeLa cells (2.5 x 10^5^ cells) transiently transfected with 4 μg pSG5 or pSG-Tax-His using TurboFect. Whole cell extracts (50 μg) were analyzed 48h later by Western blot using antibodies against MyoF, Tax (6xHis) and β-actin. (B) The graph shows quantification of band intensities of MyoF normalized to band intensities of β-actin averaged from four independent experiments. Error bars show standard deviations. (TIF) [file ppat.1011202.s001.tif]

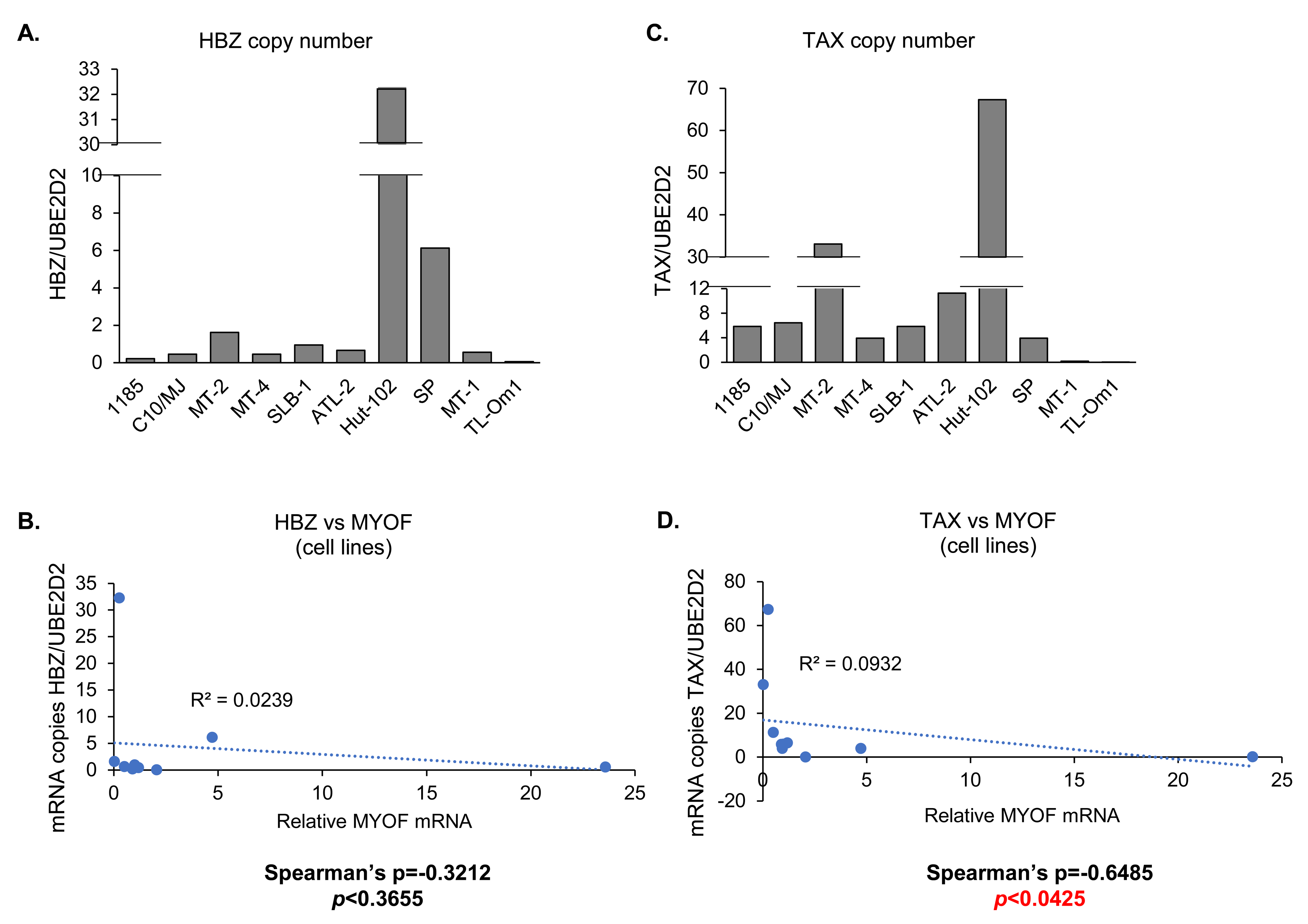

Supplement: S2 Fig — (A) and (C) hbz mRNA and tax mRNA copies per UBE2D2 mRNA copy (housekeeping gene), respectively, in HTLV-1-infected T-cell lines. (B) and (D) Linear regression analysis of relative MYOF mRNA to hbz mRNA copies and tax mRNA copies, respectively. MYOF mRNA values were normalized to that for SLB-1 cells (set to 1). A Spearman correlation test was used based on the none-normal distribution of the data. (TIF) [file ppat.1011202.s002.tif]

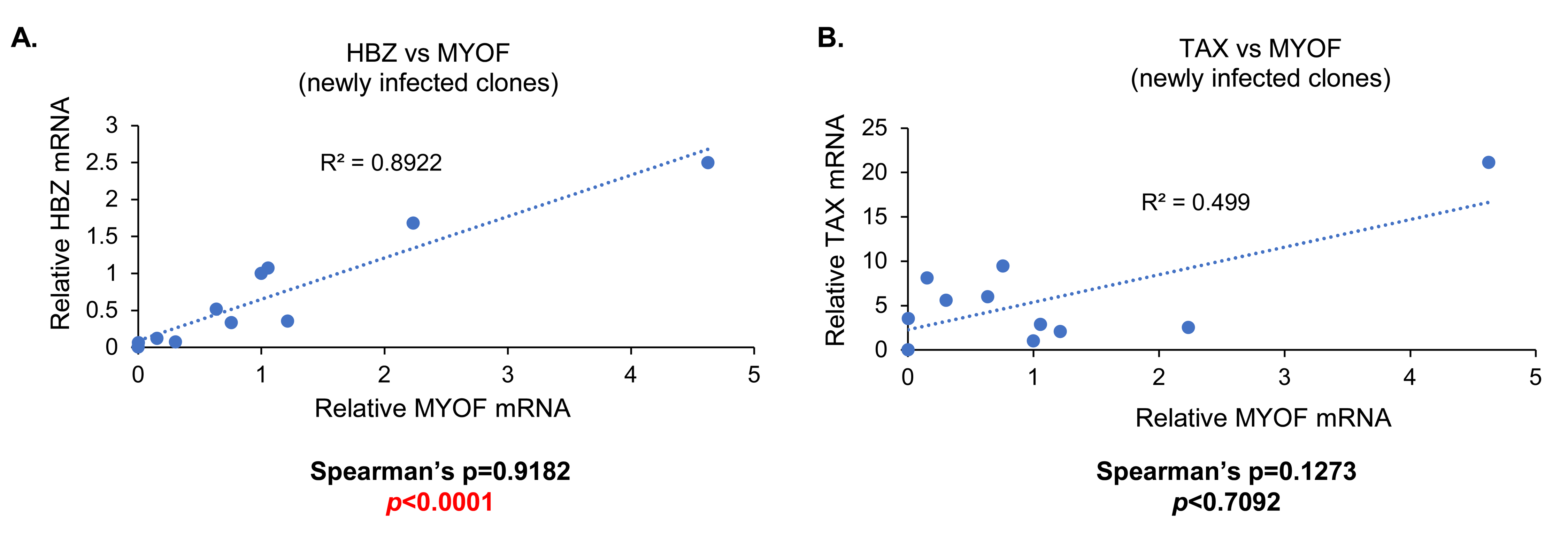

Supplement: S3 Fig — MYOF, hbz and tax mRNA levels were normalized to that for clone C15 (set to 1). A Spearman correlation test was used based on the none-normal distribution of the data. (TIF) [file ppat.1011202.s003.tif]

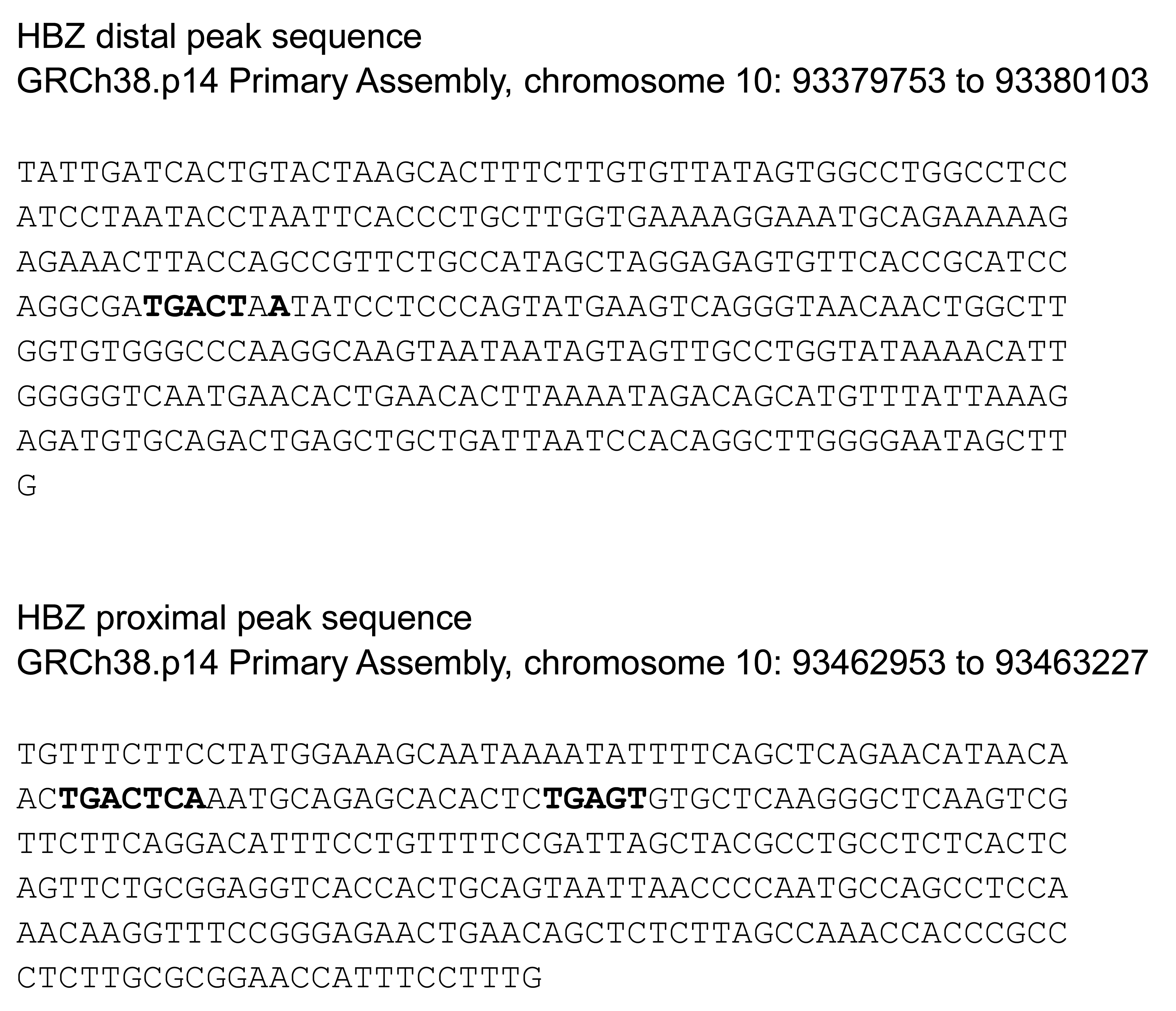

Supplement: S4 Fig — Peak sequences were determined using the UCSC Genome Browser (http://genome.ucsc.edu; [79]) with peak coordinates subsequently extrapolated to the current genome assembly. Bolded sequences denote full or partial AP-1 binding sites. (TIF) [file ppat.1011202.s004.tif]

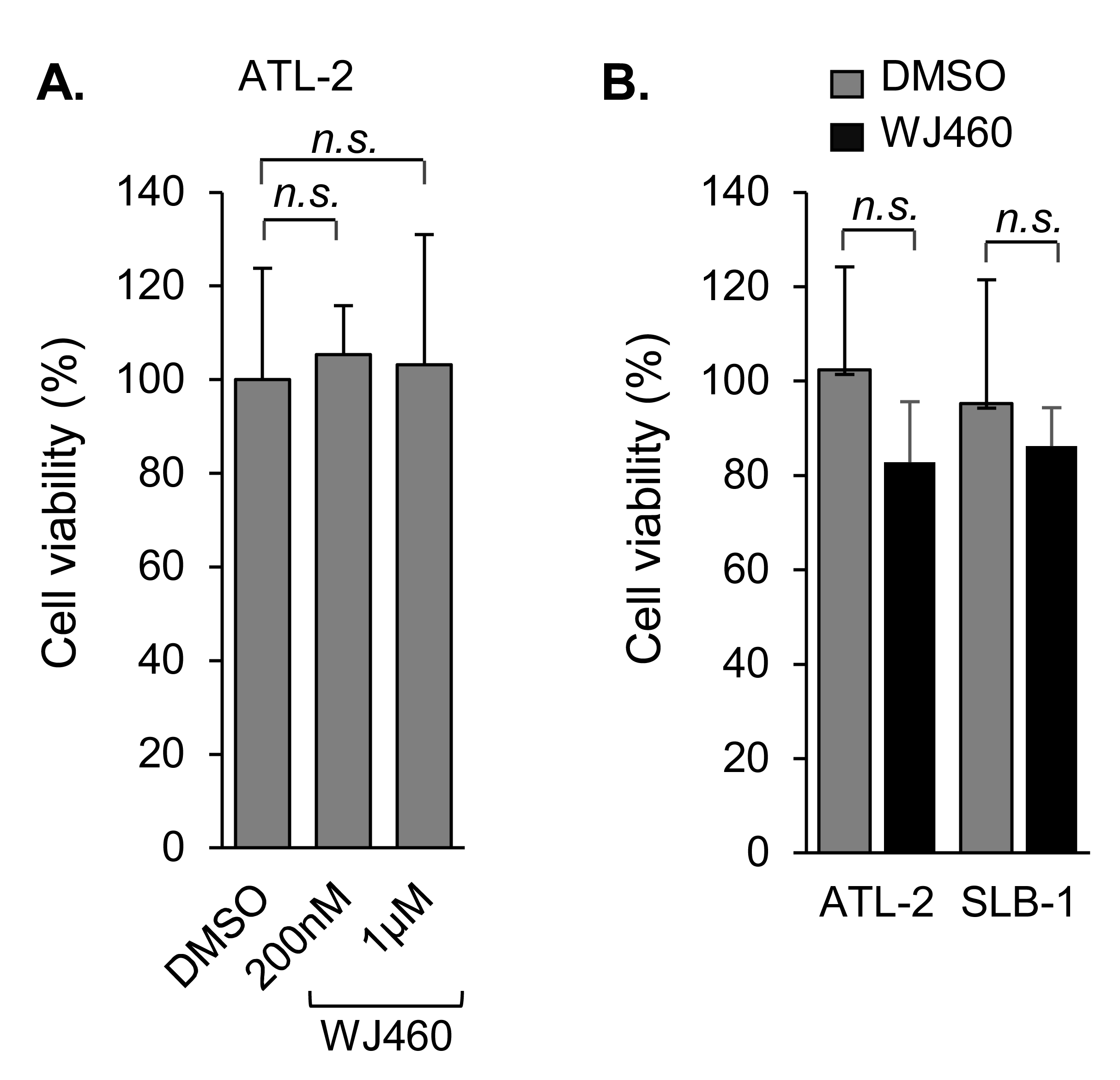

Supplement: S5 Fig — (A) ATL-2 cells were treated with DMSO or WJ460 (200 nM and 1μM) for 24 h. (B) ATL-2 and SLB-1 cells were treated with DMSO or 1μM WJ460 for 24 h. Graphs show average cell viabilities for eight (A) and six (B) replicates. Values are normalized to a single DMSO treatment replicate for each experiment/cell line. (TIF) [file ppat.1011202.s005.tif]

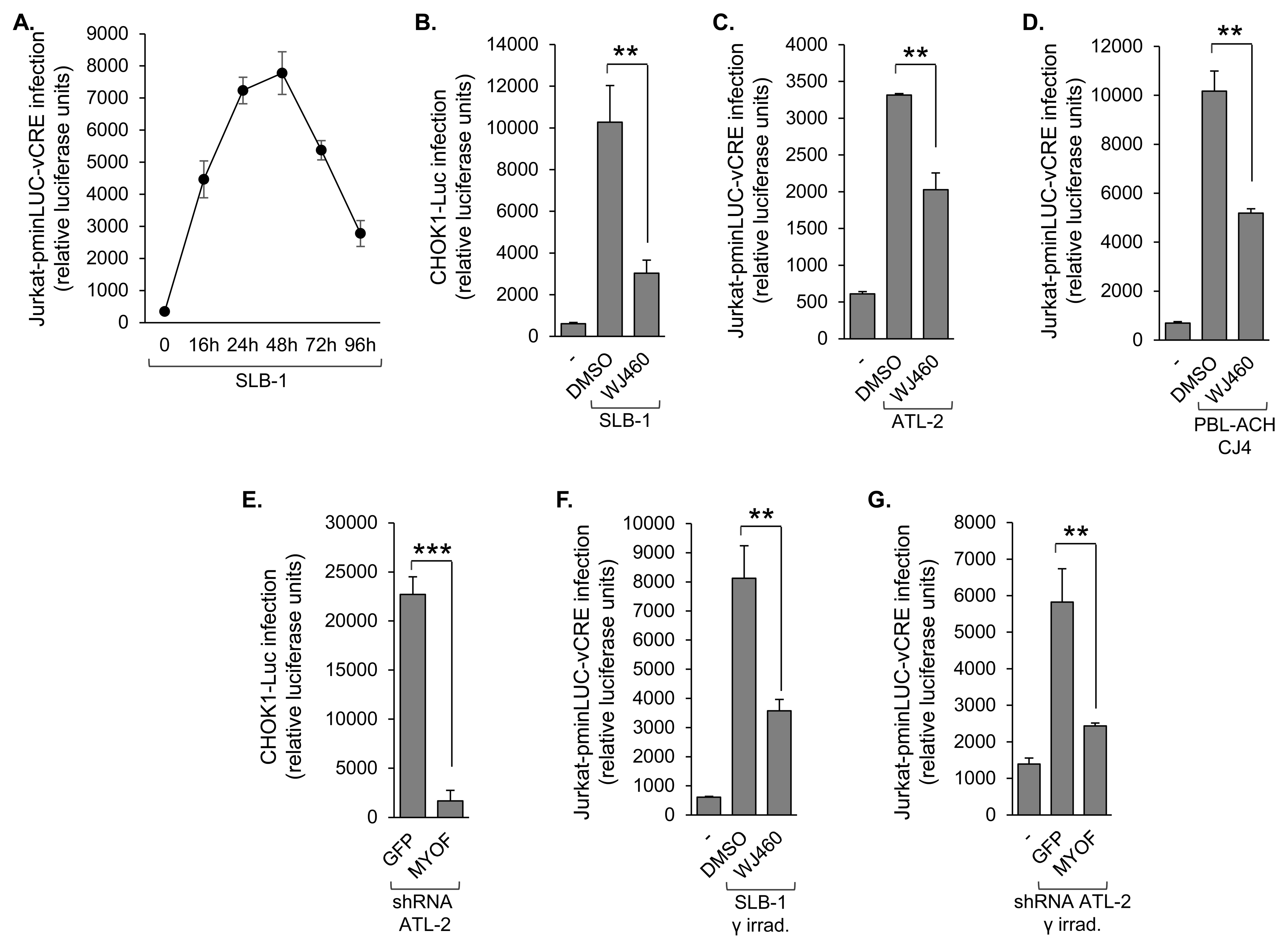

Supplement: S6 Fig — (A) SLB-1 cells were co-cultured with Jurkat-pminLUC-vCRE cells, harvested and analyzed for luciferase activity at the times indicated. The graph shows luciferase assay results average from three replicates for each time point and is representative of two independent experiments. (B) SLB-1 cells were treated with DMSO or 1 μM WJ460 prior to co-culture with CHOK1-Luc cells. (C) ATL-2 were treated with DMSO or 1 μM WJ460 prior to co-culture with Jurkat-pminLUC-vCRE. (D) The HTLV-1-immortalized primary human T-cell clone, CJ4, was treated with DMSO or 1 μM WJ460 prior to co-culture with Jurkat-pminLUC-vCRE cells. (E) ATL-2 cells stably expressing an shRNA targeting GFP or MYOF mRNA were co-cultured with CHOK1-Luc cells. (F) SLB-1 cells were treated with DMSO or 1 μM WJ460 and then irradiated (77 Gy) prior to co-culture with Jurkat-pminLUC-vCRE cells. (G) ATL-2 cells stably expressing an shRNA targeting GFP or MYOF mRNA were irradiated (77 Gy) prior to co-cultured with Jurkat-pminLUC-vCRE cells. Graphs show luciferase assay results averaged from three replicates for each condition of a single experiment; **p<0.01, ***p<0.001. Graphs (B) and (C) are representative of at least three independent experiments. Graphs (A), (D), (E), (F) and (G) are representative of two independent experiments. (TIF) [file ppat.1011202.s006.tif]

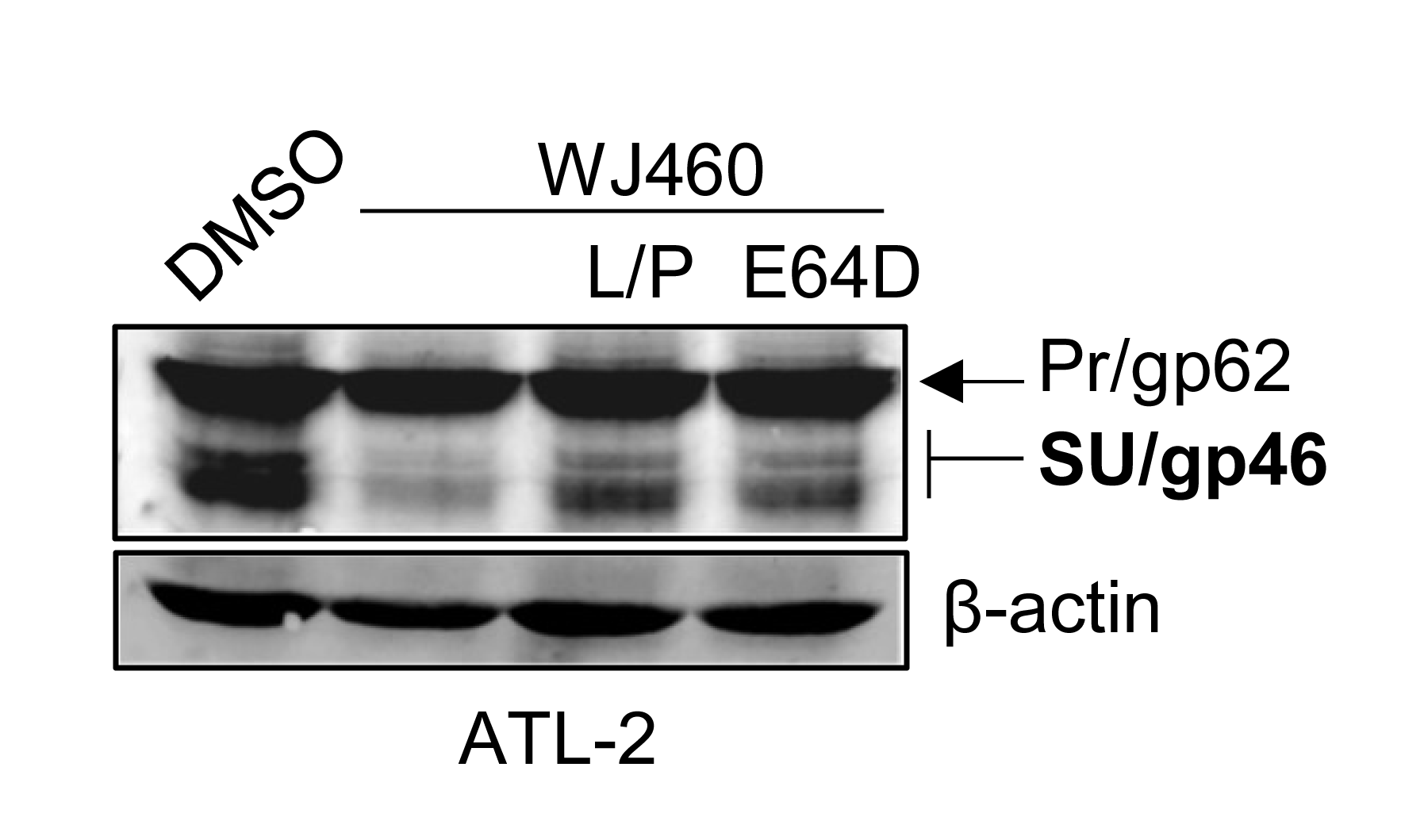

Supplement: S7 Fig — ATL-2 cells were treated with DMSO or 1 μM WJ460 with or without 5 mg/mL leupeptin (L) and 5mg/ml pepstatin A (P) or 25 μM E64D (E) for 24 h. Whole cell extracts (50 μg) were analyzed by Western blot using antibodies against β-actin and SU/Pr. (TIF) [file ppat.1011202.s007.tif]
