# Supplementary material for: HBZ upregulates myoferlin expression to facilitate HTLV-1 infection
Source: PLoS Pathog. 2023 Feb 24;19(2):e1011202. doi: 10.1371/journal.ppat.1011202 (PMC9994761; doi:10.1371/journal.ppat.1011202)
Supplement: Attachment — Submitted filename: Cover lettter resub Polakowski.pdf [file ppat.1011202.s008.pdf]

Department of Microbiology and Immunology
Brody School of Medicine | 600 Moye Boulevard
East Carolina University | Greenville, NC 27834-4354
252-744-2700 office | 252-744-3104 fax

Dear Editors,

Thank you for your positive response to our manuscript, and we would like to thank the reviewers for their positive feedback and their critical perusal of the manuscript. We have attempted to address all reviewers' recommendations. Listed below, are descriptions of revisions to the manuscript in accordance with reviewer comments. Changes are highlighted in red in the manuscript.

Responses to major issues raised by Reviewer #1:
Figure 1C: it would be benefitial to present Tax and HBZ copy numbers of the HTLV-1-infected cell lines and to present correlations with MyoF transcripts.
hbz and *tax* mRNA copy numbers normalized to those from the *UBE2D2* housekeeping gene have been determined for one set of RNA specimens, and also for the analysis, relative *MYOF* mRNA values were revaluated for this set. Correlation analyses (*hbz* versus *MYOF* and *tax* versus *MYOF*) are shown in supplementary figure 2.

Figure 1D: Can HBZ protein be detected in these cell lines?
A western blot for HBZ has been added to the figure 1D.

Figure 2B, Lines 187-188: please show correlations between HBZ expression and Tax expression, respectively, with MyoF (e.g. in supplement).
For figures 2B and 2C, correlation analyses (*hbz* versus *MYOF* and *tax* versus *MYOF*) have been done and are shown in supplementary figure 3.

Figure 5: The most important control of the experiments shown in 5 are co-cultures between non-producing C8166 cells and the reporter cells. Please include this control in the main manuscript and not only on the supplement.
The graph showing data for the negative control co-culture infection assays using C8166 cells has been moved from the supplement to the main manuscript (now figure 5D).

Response to the major issue raised by Reviewer #2:
On lines 187-188, the Authors state that there was no correlation between Tax and MOYF expression levels in their infected clones, and that this confirms that Tax is not involved in MYOF induction. This assertion should be supported by experiments in which MYOF is quantified by immunoblot in Tax-transfected cells (HeLa cells or a Tax-negative T-cell line).
MyoF levels were analyzed from HeLa cells transfected with an empty- or a Tax-expression vector. A western blot and the quantification of the data are shown in supplementary figure 1.

Responses to minor issues raised by Reviewer #1:
Figure 1B: Please show HBZ protein expression. A western blot for HBZ has been added to figure 1B.

Figures 1 E, F, 2A, 3A: These data were obtained from published data sets. Please provide reference of data set also in the respective figure legends. GEO accession numbers and corresponding references have been added to figure legends where required.

Figure 5D: This experiments was just performed twice, therefore, I would remove statistics from this experiments. For figure 5E (formerly 5D), the graph shows statistics for three replicates from a single experiment. This experiment was performed twice with the same outcome each time.

Earlier work has shown that transactivation of co-cultured reporter cells peaks at 2-4 d post co-culture (PMID: 26269171). Is this also true for the reporter cell lines used by the authors? Using our system, luciferase activity of Jurkat reporter cells peaked at 1 and 2 days, which is now shown in supplemental figure 6A.

Line 198: TSS should be introduced. Done.

Line 556: LRS should be introduced. LRS has been changed to leukocyte reduction system.

Line 581: is there a reason why Olido (dT) primers instead of random hexamer primers were used for cDNA synthesis prior to HBZ qPCR? Please explain. Oligo dT primers were used for HBZ cDNA synthesis, based on a recent report by Ma *et al.* [Proc Natl Acad Sci U S A. 2021 Apr 27;118(17)], in which *hbz* RNA was reported to be inefficiently polyadenylated and thus retained in the nucleus. We reasoned that Oligo dT cDNA would more accurately reflect HBZ mRNA destined for translation. The Materials and Methods have been modified to explain this premise, as well as to describe the procedure for mRNA copy number analysis.

Line 671: please correct: „were co-cultured". Corrected.

Figure 7E/ Line 684: Please explain: why are cells treated with protease inhibitors prior to imaging? To prevent Env degradation? Cells were indeed treated with lysosomal protease inhibitors to inhibit degradation of Env in the lysosome and thereby increase detection of Env in lysosomes. This information has been added to the Materials and Methods.

Line 713: typo: precipitate instead of precipitated. The typo has been corrected.

Line 735: reference is missing. Reference has been added.

<u>Responses to minor issues raised by Reviewer #2:</u>
The immunoblots shown in Fig. 6A-C should be accompanied by bar graphs showing differences in levels of proteins of interest (normalized against beta-actin) calculated as means from at least 3 experimental replicates. From the immunoblots shown in Fig. 6D-F, it appears that MYOF induces expression of PR/gp62; the increase in SU/gp46 is much less evident. Graphs quantifying gp46 and gp62 protein levels related to WJ460 treatment and shRNA-mediated knockdown have been added as figure 6C and 6E, respectively. As noted by the Reviewer, ectopic MyoF expression seems to be associated with an increase in gp62 in HEK293T cells. The perception that this effect is more evident than the increase in gp46 may be due to the overall higher band intensities for gp62.

Lines 695-696 and legend to Fig. 7E: more detail on confocal microscopy should be provided. Was colocalization calculated layer-by-layer? What was the thickness of the optical slice? How were the borders of the cells visualized? We added details for the confocal microscopy to the Materials and Methods. As now stated, we analyzed a single slice since many slices did not have a high proportion of pixels above background (regions where Env an/or LAMP-2 are not present) and would thus not be appropriate for a Manders' analysis. In the figure legend (7E), we defined the thickness of the optical slices.

Was the antibody used to detect Su/gp46 in immunofluorescence and flow cytometry specific for this protein, or does it also detect Pr/gp62? The antibody does recognize both SU and gp62, which is now stated in the Results section associated with the confocal microscopy. Here, we also describe the evidence indicating that SU is likely the predominant form associated with LAMP-2. Unfortunately, there is no commercially-available antibody that distinguishes between gp46 and gp62 for these experiments.

Abstract, lines 27-31. The two phrases describing HBZ's enhancement of infection through upregulation of ICAM-1 could be replaced by a single phrase similar to the one on lines 100-101; this would provide a smoother transition into the topic of the study. Done.

On line 170, replace 'ATL patient cells' with 'ATL cell lines'. The legend to Figure 2A should indicate the control used to calculate the percent reduction in MYOF transcript levels. Done for both.

Line 247: delete 'Fig.2.' Done.

In Fig. 6a, there might have been an error in image assembly - the immunoblot strip for Env proteins appears to show several images superimposed over each other. The western blot in figure 6A is from the scan and not from a superimposed image. The antibody used for this western-blot (from the NIH AIDS Research program) gave more background then the antibodies from Abcam and Santa-Cruz Biotechnology.

The colors in the graphs in Figs. 7C and 8A do not correspond to the legends. The colors of the legends in figures 7C and 8A have been changed to better identify the histograms.

On line 735, the reference for GSE94409 is missing. The reference has been added.

Line 747: References. Done.

Lines 1191-1193, 1201-1204, 1281-1282: do the graphs show the means from 3 experiments? Or is this a single experiment that is representative of a total of 3 experiments? (Also see line 1335, 'presentative'). In general, the exact number of experiments analyzed should be indicated, rather than saying 'at least 3'. The graphs show data from replicates of a single experiment. Experiments were repeated as stated and reproduced the results. We used "at least" to limit confusion/complexity in the figure legends, which are already lengthy.

Antibody C4, sc-47778 recognizes beta-actin. This should be indicated in the text (line 607) and in the immunoblot figures. Done.

Sincerely,

Isabelle Lemasson, Ph.D.
Professor
Dpt. Microbiology and Immunology
Brdody School of Medicine, ECU
